# Plant Phenotypic Traits Eventually Shape Its Microbiota: A Common Garden Test

**DOI:** 10.3389/fmicb.2018.02479

**Published:** 2018-11-06

**Authors:** Yunshi Li, Xiukun Wu, Tuo Chen, Wanfu Wang, Guangxiu Liu, Wei Zhang, Shiweng Li, Minghao Wang, Changming Zhao, Huaizhe Zhou, Gaosen Zhang

**Affiliations:** ^1^Key Laboratory of Desert and Desertification, Northwest Institute of Eco-Environment and Resources (NIEER), Chinese Academy of Sciences (CAS), Lanzhou, China; ^2^University of Chinese Academy of Sciences, Beijing, China; ^3^Key Laboratory of Extreme Environmental Microbial Resources and Engineering, Lanzhou, China; ^4^State Key Laboratory of Cryospheric Sciences, NIEER, CAS, Lanzhou, China; ^5^Conservation Institute, Dunhuang Academy, Dunhuang, China; ^6^School of Environmental and Municipal Engineering, Lanzhou Jiaotong University, Lanzhou, China; ^7^State Key Laboratory of Grassland Agro-Ecosystems, Lanzhou University, Lanzhou, China; ^8^College of Computer, National University of Defense Technology, Changsha, China

**Keywords:** plant microbiota, host specificity, plant attributes, network analysis, bacterial and fungal community, phyllosphere, rhizosphere, *Picea* spp.

## Abstract

Plant genotype drives the development of plant phenotypes and the assembly of plant microbiota. The potential influence of the plant phenotypic characters on its microbiota is not well characterized and the co-occurrence interrelations for specific microbial taxa and plant phenotypic characters are poorly understood. We established a common garden experiment, which quantifies prokaryotic and fungal communities in the phyllosphere and rhizosphere of six spruce (*Picea* spp.) tree species, through Illumina amplicon sequencing. We tested for relationships between bacterial/archaeal and fungal communities and for the phenotypic characters of their plant hosts. Host phenotypic characters including leaf length, leaf water content, leaf water storage capacity, leaf dry mass per area, leaf nitrogen content, leaf phosphorous content, leaf potassium content, leaf δ^13^C values, stomatal conductance, net photosynthetic rate, intercellular carbon dioxide concentration, and transpiration rate were significantly correlated with the diversity and composition of the bacterial/archaeal and fungal communities. These correlations between plant microbiota and suites of host plant phenotypic characters suggest that plant genotype shape its microbiota by driving the development of plant phenotypes. This will advance our understanding of plant-microbe associations and the drivers of variation in plant and ecosystem function.

## Introduction

Microbes associated with leaves and roots have potentially beneficial effects on the health, fitness and evolution of the host, to the extent that different determinants on plant microbiota have been quantified in previous studies (Bulgarelli et al., 2013). The host genetic-based traits (Wieland et al., 2001; Yang et al., 2001; Timms-Wilson et al., 2006; Opelt et al., 2007; Schweitzer et al., 2007; Whipps et al., 2008; Redford et al., 2010; Bodenhausen et al., 2014) and environmental factors (*e.g.*, the environmental pool of colonizers, sites, temperature, and water) (Bogino et al., 2013; Maignien et al., 2014; Müller et al., 2016) can influence the community structure of the microbiota that is established on the host. Generally, the influence on microbiota by plant genotype, namely host effects, can be overwhelmed by environmental factors (Bulgarelli et al., 2015). The host genetic control of the microbiome is evident in some studies (Adams and Kloepper, 2002; Bálint et al., 2013; Sapkota et al., 2015; Wagner et al., 2016). For example, Sapkota et al. (2015) suggested that plant genotype at the species level reported 43% variance in the total fungal community dataset. However, the available data only reported the effect in general terms, and the mechanisms initiated by plant genotype are not well characterized. Therefore, specific plants genotype-driven factors that cause this difference need to be quantitatively evaluated.

Both host plant and environmental factors can drive the development of plant phenotypes and the assembly of plant microbiota. Environmental factors could affect the leaf and root communities indirectly by causing plasticity of plant functional traits that underlie the microbiome (Wagner et al., 2016). Studies have shown that microbial biodiversity is a trait forming part of the extended phenotype of the host plant (Whitham et al., 2003; Agler et al., 2016). Previous studies have reported significant correlations between microbial communities and plant phenotypic variations. In field studies, Laforest-Lapointe et al. (2016) demonstrated that host species identities, such as wood density and leaf nitrogen content, drive the temperate tree phyllosphere bacterial communities. Similar results were also found in fungal communities, which were more impacted by leaf physiological characters (Unterseher et al., 2016). Previous studies have also shown that leaf anatomical characters (De Costa et al., 2006) and the levels of leaf soluble carbohydrates, calcium, and phenolic compounds (Hunter et al., 2010) significantly influence bacterial community structure. In the above studies, plant phenotypes are controlled simultaneously by its genetic makeup and environmental stress. While in the common garden with consistent environmental stimulation, the differences of plant phenotypes are ultimately driven by its genotype. Multiple evidence suggests that the presence of microbes can alter plant phenotypic plasticity in a broad range of traits (Partida-Martínez and Heil, 2011; Goh et al., 2013). Hence, it is reasonable to think that the correlation between plant phenotypic characters and microbial taxa is a consequence of plant genotype effects on the microbiota.

It is therefore worth pondering if a plant’s genetic control influences all taxa of the microbiota. In the study conducted by Knief et al. (2010), the most significant differences in the *Methylobacterium* communities were observed at those sites where *Arabidopsis thaliana* plants showed higher phenotypic variation. However, there is still a poor understanding of this microbiome – the group of bacterial or fungal taxa that is responsive to the effects derived from the plant. The importance of plant genetics or genetic-based traits to the whole microbial communities is well studied (De Costa et al., 2006; Yadav et al., 2008; Reisberg et al., 2012), yet at a finer taxonomic level, little is known about the co-occurrence interrelations between individual microbial dwellers and multiple plant phenotypic identities. There is also no clear difference between the effect of prokaryotic and eukaryotic microbes in general. We hypothesize that plant species actually affect some members of the inhabiting microbes or some groups of the inhabiting microbes, instead of affecting all the taxa, because some bacteria that exist in the community are not the results of selection by the host but by stochastic process. In turn, not all microbes respond to the phenotypic variations. Further, plant species may not have the same degree of effect on every member or group of microbial communities.

In order to disentangle if plant genotypes structure the leaf and root microbiomes, we investigated the potential associations of host phenotypes with microbial genera of spruce (*Picea* spp.). Our results showed that this is a poorly studied evergreen coniferous tree species with around 35 species in the world (Stadler and Müller, 1996; Menkis et al., 2015). They were of tremendous socioeconomic importance. For example, forestry in the Nordic countries is based on coniferous trees [*e.g.*, Norway spruce *P. abies* (L.) Karst.] (Schlyter et al., 2006). The Qinghai spruce (*P. crassifolia*) is the dominant coniferous species of the Qilian Mountains (Zhao et al., 2006) in China. The importance of forestry in the national and rural economy is fundamental. China has the largest numbers of spruce (*Picea* spp.) with about 18 species (seven endemic, two introduced) (Fu et al., 1999). In this study, we performed an experiment in a common garden to exclude the influence of environmental drivers. The trees are exposed to similar initial conditions, and, thus, the plant species identity *per se* governing its associated microbiota structure can be observed.

## Materials and Methods

### Seeds, Seedling Preparation, and Growth of the Spruce

The six spruce species (genotype groups) were grown at a ‘common garden’ in the Yuzhong campus of Lanzhou University (GPS coordinates: 104.1562°E, 35.93907°N) in Lanzhou City, Gansu Province, China. They were *P. likiangensis* var. *rubescens* (PL), *P. smithiana* (PS), *P. abies* (PA), *P. crassifolia* (PC), *P. koraiensis* (PK), and *P. wilsonii* (PW). The common garden was located in the northwest of the Loess Plateau, the middle part of Gansu Province, which is 46 km from Lanzhou city. Forty years (1955–2005) of records showed that the climate of this location is semiarid and the mean annual precipitation is low (381.8 mm), nearly 78% of rainfall events are below 10 mm and 36% of precipitations are below 5 mm, and the mean annual temperature is 6.57°C and droughts are a common occurrence. The average annual pan evaporation is 930.6 mm. The soil in the region, which is 2 m deep, is the clay loam of Loess origin with a bulk density ranging from 1.38 to 1.45 g cm^-3^, and the water holding capacity of the soil is 21.18%. The results are supported by Wang et al. (2008).

The spruce species were bred by seeds. Before planting, dry seeds of these species were immersed in tap water for 24 h, at room temperature. Later, the visibly swollen ones were selected and surface-sterilized by immersion in 0.5% potassium permanganate (KMnO_4_) solution for 30 min, followed by three washes with sterile tap water. In the early year of 2006, spruce seedlings were bred in Xiaolongshan forest area (GPS coordinates: 106.5575°E, 34.1186°N) in Tianshui City, Gansu Province, China. The climate of this location is warm with humid and semi-humid continental monsoon. It is characterized by a long-term average annual temperature of 7–12°C and an average annual precipitation of 600–900 mm. The annual average relative humidity is 65–75% and the annual average evaporation is 1300–1600 mm. The soil in the area is dominated by mountain cinnamon soils and brown forest soils listed in the Chinese Soil Taxonomy (CST). The information was obtained from the Xiaolongshan Forestry Bureau of Gansu Province. After 3 years of growth, the seedlings were transplanted into the common garden in 2009.

Use of a ‘common garden’ experiment in this study provided an opportunity to minimize potential confounding effects of differences in climate, time, soil, topography, and land use (Reich et al., 2005). The year-to-year variation in soil conditions resulted from the development of spruce populations. When sampled, all the trees were 7 years old. In each phyllospheric microbial sample, 10 g/tree of needles and shoots were collected from multiple canopy positions and mixed into a 100 mL plastic centrifuge tube containing 50 mL sterile phosphate buffer. The tube was agitated on a vortex for 10 s and repeated for six times, and then the buffer was filtered through an EMD Millipore Sterivex-GV Polyvinylidene Fluoride 0.22 μm filter (Millipore, Billerica, MA, United States). Later, the filter was placed in a sterile screw-cap tube and frozen at -80°C before processing.

### Measurement of Host Attributes and Near-Root Soil Characteristics

Data on host plant phenotypic traits were obtained for all species according to the previously collected data from the State Key Laboratory of Grassland Agro-Ecosystems, School of Life Sciences, Lanzhou University. Traits related to leaf physiological characters included leaf length (LL), leaf water content (SWC), leaf water storage capacity (Cleaf), and leaf dry mass per area (LMA). Leaf constituents included the content of total nitrogen (LTN), phosphorous (P), potassium (K), and δ^13^C values (δ^13^C). Photosynthesis-related traits included stomatal conductance (Cond), net photosynthetic rate (Photo), intercellular carbon dioxide concentration (Ci), and transpiration rate (Trmmol).

For the near-root soils, five soil cores, which were 4.5 cm in diameter and 10 cm deep, were collected radially from outward the tree with 15 cm distances, mixed to form one composite soil sample. The samples were collected manually using a stainless steel shovel (type: 10 cm × 8 cm) and were surface-disinfected using alcohol between sampling and planting and between different species. We carefully removed the fine roots from the soil samples and stored the samples in separate sterile plastic bags, which were then rapidly frozen under ice for transportation. Each soil sample was divided into two parts, one for DNA extraction (frozen at -20°C) and the other for physical and chemical properties. The soil water content (MC) was determined gravimetrically after the soil was dried in an oven at 105°C for 12 h (Liu et al., 2010). The total carbon (TC), organic carbon (OC), and total nitrogen contents (STN) were quantified with an automatic element analyzer (Elementar Vario EL, Germany) (Liebner et al., 2009). The soil potential of hydrogen (pH) was measured in a 1:2.5 soil/H_2_O suspension using a waterproof pH/ORP meter. The total dissolved solids (TDS), electric conductivity (EC) and the total soil salinity (Salinity) were measured with a conductivity/TDS meter (Atekwana et al., 2004).

### DNA Extraction, PCR Amplification, and High-Throughput Sequencing

Genomic DNA of the soil and phyllosphere microbes was extracted using the PowerSoil DNA Kit (MoBio Laboratories, Carlsbad, CA, United States) according to the manufacturer’s instructions. The quality and quantity of extracted DNA were assessed by NanoDrop ND-1000 spectrophotometer (NanoDrop Technologies, Wilmington, DE, United States). The PCR amplification was done according to the instructions in the protocol described on the website of the Earth Microbiome Project^1^. In brief, primers 515F-806R (Bates et al., 2011) and ITS1-ITS2 (White et al., 1990) were used to target partial fragments of the 16S rRNA gene and the fungal internal transcribed spacer (ITS) gene, respectively. Amplicons were extracted from 2% agarose gels and purified using the AxyPrep DNA Gel Extraction Kit (Axygen Biosciences, Union City, CA, United States) according to the manufacturer’s instructions and quantified using QuantiFluor^TM^-ST (Promega, Madison, WI, United States). The purified amplicons were pooled in an equimolar concentration and sequenced in a paired-end manner (2 bp × 300 bp) on an Illumina MiSeq platform (Allwegene, Beijing, China) according to the standard protocols.

### Data Processing

The raw FASTQ files were demultiplexed and quality-filtered using QIIME software (version 1.8.0^2^; Caporaso et al., 2010) depending on the following criteria: (i) The 300-bp reads were truncated at any site that obtained an average quality score of <20 over a 10-bp sliding window, and the truncated reads shorter than 50 bp were discarded; (ii) exact barcode matching, two nucleotide mismatch in primer matching, and reads containing ambiguous characters were removed; (iii) only overlapping sequences longer than 10 bp were assembled according to their overlapped sequence. The reads that could not be assembled were discarded. Operational taxonomic units (OTUs) with 97% similarity cutoff were clustered using UPARSE v.7.1 (Edgar, 2013), and chimeric sequences were removed using UCHIME (Edgar et al., 2011). The taxonomy of each 16S rRNA gene and ITS sequence was analyzed against the Silva (Release 119^3^; Quast et al., 2012) and UNITE (Release 7.0^4^; Kõljalg et al., 2013) database at a confidence threshold of 70%, respectively. The rarefaction analysis based on Mothur v.1.21.1 software (Schloss et al., 2009) was conducted to reveal the diversity indices, including the ACE, Chao1, Shannon, Simpson, and coverage indices. The phyllosphere and rhizosphere metagenome raw sequence reads have been uploaded in the NCBI Trace Archive and the NCBI Sequence Read Archive repositories (BioProject PRJNA 454087).

### Data Analyses

To explore the variation in effects of plant species on richness, evenness, and diversity of microbial communities, we used two-way analysis of covariance (ANCOVA) models (Maalouf et al., 2012). Principal coordinates analysis (PCoA) plots were generated to compare the composition of bacterial/archaeal and fungal community structure among the different treatments. Permutational multivariate analysis of variance (PERMANOVA) on the Bray-Curtis metric produced by PCoA analysis was performed to test the significant difference in community composition among the treatments. To examine co-occurrence networks in microbial communities, network analysis was conducted on soil/leaf properties and microbial data using the Spearman’s correlation, which was analyzed with the psych package in R (Revelle, 2012). Relationships between edaphic factors and biotic variables that were significant at *p* < 0.05 were then visualized in CYTOSCAPE version 3.2.0 (Shannon et al., 2003).

## Results

### Variations of the Leaf and Near-Root Soil Characteristics

As shown in Table 1, for the leaf constituents, LTN ranged from 10.54 to 14.14 g kg^-1^, K ranged from 3.09 to 6.95 g kg^-1^, P ranged from 1.11 to 1.68 g kg^-1^, and δ^13^C ranged from -27.14 to -23.67. Leaf physiological related characters showed significant intergroup variation. The LL ranged from 1.66 to 3.57 cm, and PS showed substantially greater leaf length than the rest. The LMA ranged from 21.77 to 26.42 g m^-2^. The highest and lowest SWC were shown by PS (2.08 g g^-1^) and PA (1.26 g g^-1^). The Cleaf ranged from 21.77 to 26.42 g m^-2^. Photosynthesis related characters – photo, Cond, Ci, and Trmmol – also showed highly significant variation between species.

**Table 1:** Host attributes information of the six tree species and near-root soil characters used in this study.

	PL	PS	PA	PC	PK	PW
**Leaf attributes**						
LL (cm)	1.66 ± 0.05	3.57 ± 0.12	2.37 ± 0.08	2.54 ± 0.1	2.05 ± 0.12	1.55 ± 0.06
LMA (g m^-2^)	22.7 ± 2.38	23.6 ± 0.93	24.87 ± 0.34	26.42 ± 1.65	24.1 ± 1.29	21.77 ± 1.70
SWC (g g^-1^)	1.53 ± 0.03	2.08 ± 0.16	1.26 ± 0.07	1.29 ± 0.03	1.27 ± 0.02	1.55 ± 0.02
Cleaf (mol m^-2^ M Pa^-1^)	2 ± 0.06	1.62 ± 0.06	1.06 ± 0.04	1.52 ± 0.03	1.64 ± 0.06	2.08 ± 0.23
LTN (g kg^-1^)	14.14 ± 0.89	13.52 ± 0.11	12.72 ± 0.23	11 ± 0.07	10.89 ± 0.36	10.54 ± 0.64
P (g kg^-1^)	1.27 ± 0.03	1.45 ± 0.02	1.54 ± 0.05	1.68 ± 0.02	1.46 ± 0.05	1.11 ± 0.02
K (g kg^-1^)	3.09 ± 0.13	6.95 ± 0.21	4.83 ± 0.31	3.79 ± 0.25	3.65 ± 0.21	4.00 ± 0.08
δ^13^C	–25.25 ± 0.88	–24.83 ± 0.16	–23.67 ± 0.10	–24.23 ± 0.26	–25.45 ± 0.36	–27.14 ± 0.92
Photo (μmol CO_2_ m^-2^ s^-1^)	10.21 ± 0.38	7.09 ± 0.2	8.07 ± 0.32	8.60 ± 0.41	8.91 ± 0.24	9.57 ± 0.16
Ci (μmol CO_2_ mo^l-1^)	187.61 ± 3.87	194.58 ± 3.61	189.62 ± 3.31	171.59 ± 2.89	195.68 ± 6.32	204.72 ± 2.06
Trmmol (mmol H_2_O m^-2^ s^-1^)	2.53 ± 0.05	3.00 ± 0.15	3.59 ± 0.14	2.60 ± 0.13	3.14 ± 0.23	4.17 ± 0.11
Cond (mmol m^-2^ s^-1^)	111.9 ± 5.92	79.512 ± 0.04	96.857 ± 0.05	85.87 ± 6.39	105.55 ± 8.39	118.5 ± 10.28
**Soil characters**						
STN (%)	0.10 ± 0.007	0.12 ± 0.009	0.10 ± 0.007	0.09 ± 0.001	0.11 ± 0.002	0.09 ± 0.004
TC (%)	2.85 ± 0.15	3.01 ± 0.10	2.85 ± 0.15	2.57 ± 0.02	3.06 ± 0.06	2.62 ± 0.06
OC (%)	1.53 ± 0.07	1.57 ± 0.04	1.53 ± 0.07	1.39 ± 0.006	1.23 ± 0.21	1.41 ± 0.03
pH	7.38 ± 0.12	7.42 ± 0.10	7.38 ± 0.12	7.54 ± 0.05	7.2 ± 0.06	7.45 ± 0.10
TDS (mg L^-1^)	51.92 ± 4.03	43.09 ± 0.35	49.9 ± 0.55	41.9 ± 0.73	56.88 ± 4.1	48.69 ± 4.21
EC (S m^-1^)	104.02 ± 8.13	86.29 ± 0.70	100.02 ± 1.08	83.97 ± 1.48	114.18 ± 8.17	97.56 ± 8.47
Salinity (%)	0.01 ± 0.002	0.003 ± 0.002	0.013 ± 0.004	0	0.01 ± 0.002	0.01 ± 0.002
MC (%)	11.13 ± 0.73	22.77 ± 0.42	13.5 ± 0.45	19.1 ± 1.07	21.57 ± 0.59	11.08 ± 0.76


*Sources for functional trait information are described in the main text. Data are showed with Mean ± SE. LL, leaf length; LMA, leaf dry mass per area; SWC, leaf water content; Cleaf, leaf water storage capacity; LTN, content of leaf total nitrogen; P, phosphorous content in leaf; K, potassium content in leaf; δ^*13*^C, leaf δ^*13*^C values; Cond, stomatal conductance; Photo, net photosynthetic rate; Ci, intercellular carbon dioxide concentration; Trmmol, transpiration rate; STN, content of soil total nitrogen; TC, total carbon in soil; OC, organic carbon in soil; TDS, total dissolved solids in soil; EC, soil electric conductivity; Salinity, total soil salinity; and MC, soil moisture content.*

For variables of near-root soil, only MC showed highly significant intergroup variation, the values ranged from 11.08 to 22.77%, PS showed the highest soil moisture content while PW showed the lowest. The STN ranged from 0.09 to 0.12%, TC ranged from 2.57 to 3.06%, OC ranged from 1.23 to 1.57%, TDS ranged from 41.9 to 154.12%, EC ranged from 83.97 to 309.02 S m^-1^, the Salinity ranged from 0 to 0.03, and the soil pH ranged from 6.93 to 7.54.

### Phyllosphere and Near-Root Soil Microbial Communities Associated With Spruce Trees

Illumina sequencing yielded a total of 2,533,243 and 2,260,529 raw reads of 16S rRNA gene sequences and ITS gene sequences, respectively. The sequences that were shorter or longer than the expected amplicons size and chimeras were also removed. After quality filtering (the taxa of Cyanobacteria and Mitochondria were also filtered), 1,650,019 and 2,116,788 valid sequences were clustered into 7,121 prokaryotic and 2,038 fungal OTUs at 97% sequence identity level, respectively.

**FIGURE 1:**
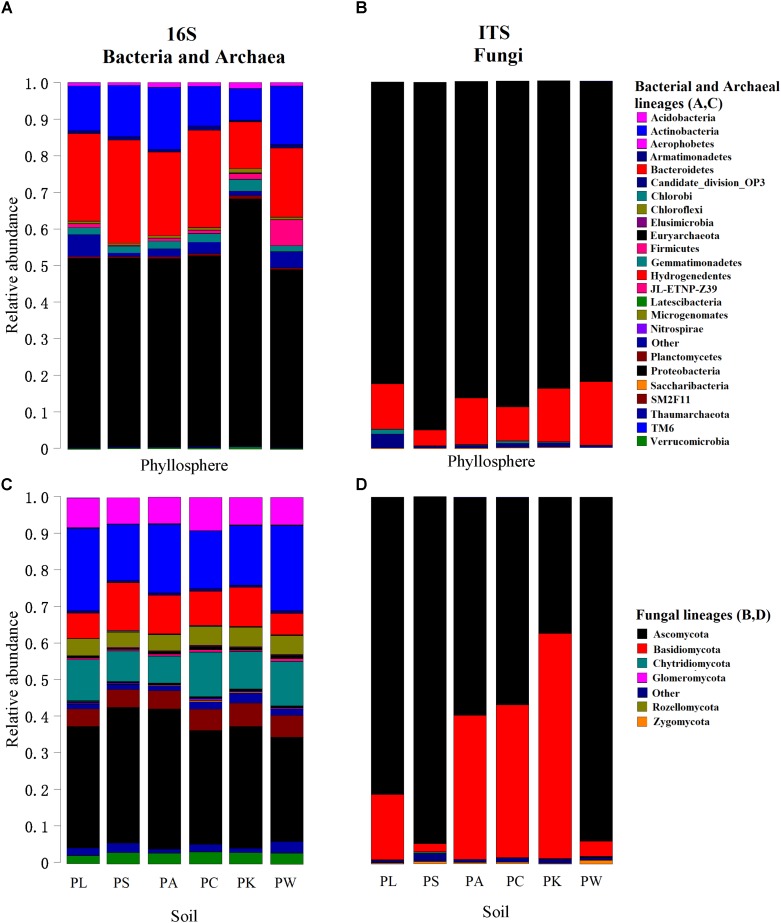
Relative abundances of major bacterial/archaeal **(A,C)** and fungal **(B,D)** phyla for each leaf or root sample. The phyla are shown in alphabetical order.

Overall, we identified 35 bacterial and 3 archaeal phyla in all investigated samples. The OTUs belonging to the bacterial phyla Proteobacteria, Actinobacteria, Bacteroidetes, Gemmatimonadetes, Acidobacteria, Planctomycetes, and Chloroflexi comprised more than 91% of the relative abundance (RA) in the bacterial communities (Figures 1A,C). In the case of fungi, we detected seven fungal phyla in our dataset, with the phylum Ascomycota being the most abundant (more than 76% of the total RA) in all samples (Figures 1B,D). At the genus level, there were 862 bacterial/archaeal and 386 fungal genera, of which 32 and 45% taxa could not be assigned to known bacterial/archaeal and fungal genera, respectively.

### Diversity Indices Variations and ANCOVA Analysis With Leaf and Near-Root Soil Characteristics

When the phyllosphere and near-root soil microbial communities of the six groups were analyzed at a 3% dissimilarity level, the microbial diversity (represented by the Shannon index), species richness (represented by the Chao1 index), and evenness (represented by the Simpson index) were estimated. The three diversity indices were used to compare microbial diversity in the communities associated with host and near-root soil characteristics.

**FIGURE 2:**
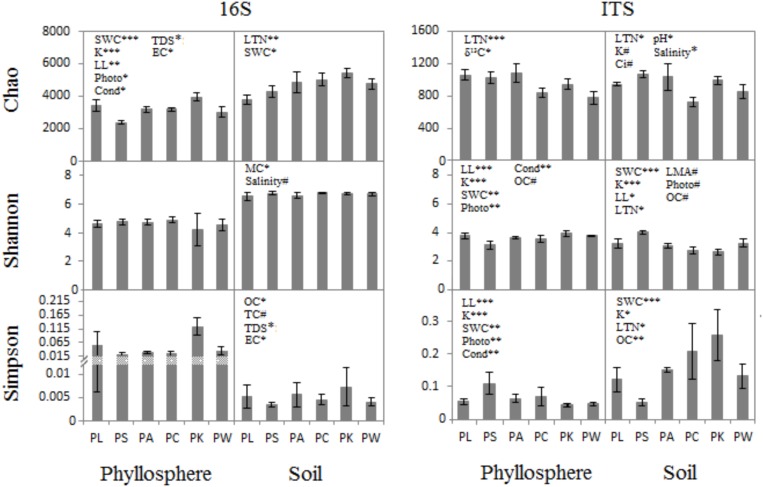
ANCOVA analysis for diversity indices (Mean ± SE) with host and near-root soil characteristics. ^∗^*p* < 0.05; ^∗∗^*p* < 0.01; ^∗∗∗^*p* < 0.001; ^#^*p* < 0.1.

The ANCOVA analysis showed that there were correlations between the host/soil factors with both the bacterial/archaeal and fungal communities (Figure 2 and Supplementary Tables S1, S2). For example, five host characters – SWC, K, LL, Photo, and Cond – had a correlation with prokaryotic richness in phyllosphere (*F* = 19.16, *p* = 0.0005; *F* = 19.9, *p* = 0.0004; *F* = 9.303, *p* = 0.0076; *F* = 6.623, *p* = 0.0204; *F* = 5.04, *p* = 0.0393; Supplementary Table S1), whereas there was no significant correlation of host factors for prokaryotic evenness (Supplementary Tables S1, S2). Host attributes were correlated with microbial communities both in phyllosphere and near-root soil. While the characters of the near-root soil only had a correlation with microbial communities in soil, OC showed a significant correlation between them (*F* = 3.68–9.15, *p* = 0.008–0.073; Supplementary Table S2). The SWC was a highly significant driver of fungal community structure both in soil (*F* = 5.70–49, *p* < 0.05; Supplementary Table S2) and phyllosphere (*F* = 5.70–49, *p* < 0.01; Supplementary Table S1). Photo and Cond were much more highly correlated with leaf fungal communities (*F* = 12.99–15.49, *p* = 0.001–0.002; Supplementary Table S1) than with prokaryotic communities (*F* = 5.04–6.62, *p* = 0.02–0.039; Supplementary Table S1). They were also much more highly correlated with leaf fungal communities (Shannon, *F* = 13.93, 14.81, *p* < 0.01; Simpson, *F* = 12.99, 15.49, *p* < 0.01; Supplementary Table S1) than with prokaryotic communities (only Chao significant, *F* = 6.62, 5.04, *p* < 0.05; Supplementary Table S1). Other characters, such as LTN, K, δ^13^C, soil pH, TDS, EC, and MC, were correlated with the bacterial/archaeal or the fungal communities either in phyllosphere or in near-root soil to different degrees (Supplementary Tables S1, S2).

### Species Composition and Host Specificity

**FIGURE 3:**
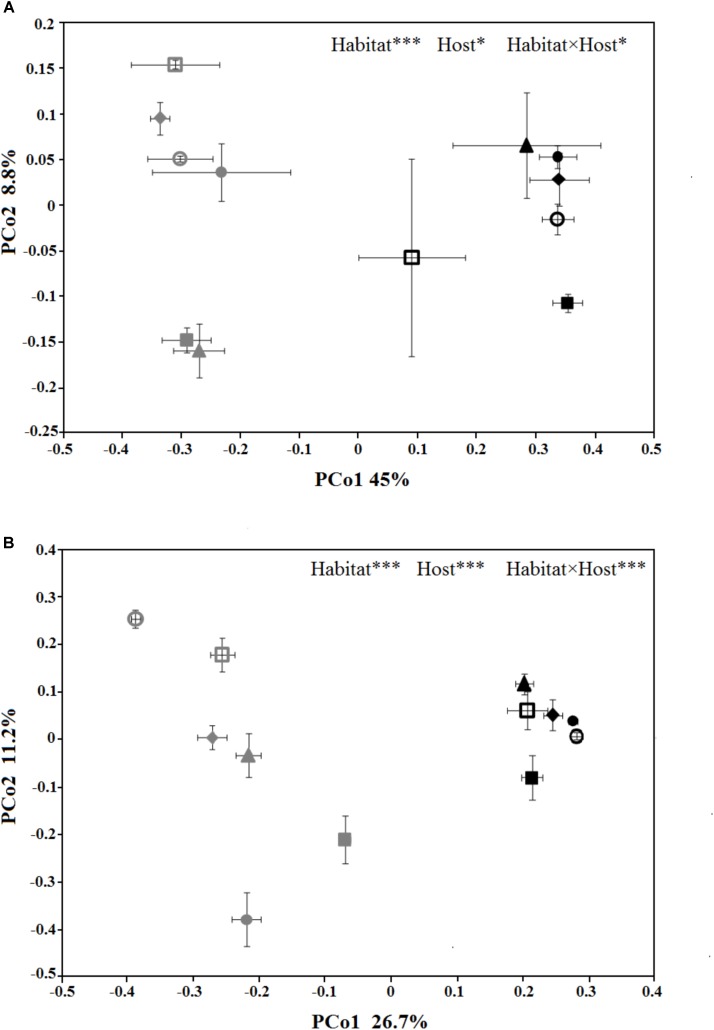
Principal coordinates analysis (PCoA) ordination of the bacterial/archaeal **(A)** and the fungal **(B)** communities, associated to the six spruce species phyllosphere and near-root soil. The values are means of three replicate samples plus SE for each host type. The symbols are as follows: triangles = PL; solid squares = PS; solid circles = PA; empty circles = PC; empty squares = PK; and diamonds = PW. The full name of each plant species is reported into the text. The symbols color-gray and black, indicate microbial communities in phyllosphere and soil, respectively. The results of relationships between microbial community structure and host species identity in the PERMANOVA are given in the higher right of each panel: ^∗^*p* < 0.05; ^∗∗^*p* < 0.01; ^∗∗∗^*p* < 0.001.

The PCoA plots were generated to compare the composition of the microbial community among different treatments (Figure 3). In PCoA plot of the bacterial/archaeal community (Figure 3A), PCo1 explained 45% of the variances and clearly divided the samples from phyllosphere and near-root soil, while PCo2 explained 8.8% of the variances and mainly differentiated the host species. As for fungal community (Figure 3B), PCo1 explained 26.7% of the variances and also distinguished the samples from phyllosphere and soil, and PCo2 explained 11.2% of the variances and also differentiated the host species. Additionally, PCoA of the fungal community showed that the variances among the samples from phyllosphere were higher than that from the soil. The PERMANOVA results indicated that habitat effects, namely phyllosphere versus rhizosphere, were highly significant in both communities (*R*^2^ = 0.42, *p* < 0.001; *R*^2^ = 0.24, *p* < 0.001; Supplementary Table S3). As for the effects from host species, it is significant in the bacterial/archaeal community (*R*^2^ = 0.13, *p* < 0.05; Supplementary Table S3) and highly significant in the fungal community (*R*^2^ = 0.26, *p* < 0.001; Supplementary Table S3). The interaction between habitat and host species effects is also significant in the bacterial/archaeal community (*R*^2^ = 0.13, *p* < 0.05; Supplementary Table S3) and highly significant in the fungal community (*R*^2^ = 0.25, *p* < 0.001; Supplementary Table S3).

### Connectedness and Co-occurrence Interrelations in Networks

**FIGURE 4:**
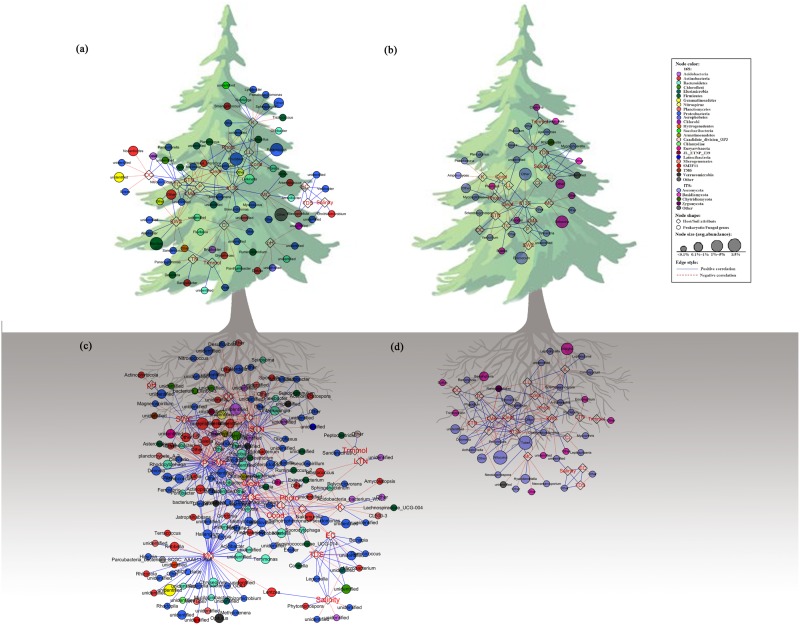
Network analysis showing connectedness between prokaryotic/fungal communities and host/soil factors in the phyllosphere **(a,c)** and near-root soil **(b,d)** across the plant holobiont. The blue solid lines represent significantly strong (>0.6) positive linear relationship, red dashed lines represent strong (<–0.6) negative linear relationship. The variables in hexagon boxes show various soil attributes and leaf characters.

Individual bacterial and fungal taxa were sensitive to the tested characters at multiple taxonomic levels, with a stable trend from the family level to OTUs (Supplementary Figure S1). The network was performed at the genus level, based on the stable trend. The co-occurrence network was compartmentalized into some clusters connecting plant characters and microbes. As for prokaryotic communities (Figures 4A,C and Supplementary Tables S4, S5), Cleaf was associated with the maximum number of nodes with most of them belonging to Proteobacteria. The LMA, P, K, and δ^13^C were also mostly associated with Proteobacteria, and Cond and Ci were mostly associated with Bacteroidetes. While LTN and Trmmol were associated with only one or two nodes, the other attributes were associated with a wide range of nodes. As for fungal communities (Figures 4B,D and Supplementary Tables S6, S7), Ascomycota is the main phylum that was associated with nearly all attributes. The phylum Basidiomycota was associated with a few nodes, for example, STN and Trmmol. The genera of the microbial communities showed different responses to phenotypes. For example, in the phyllosphere, the bacterial genus *Lactobacillus* was related to LL, Photo, and Cond, while *Brevundimonas* was related to Ci and δ^13^C. For phenotypes, such as LTN, correlated to the genera of *Sanguibacter*, *Desemzia*, and *Panacagrimonas*, indicating that one specific trait was related to some specific microbial taxa. Similar results were obtained from the ANCOVA analysis (Supplementary Figure S2). Although many of the prokaryotic and fungi analyzed in this study were unable to be identified at the genus level, the detected network modules involved all the OTUs. Taxonomic names such as “other” and “unidentified” (has no assignment of affiliation at the relevant taxonomic level) were also shown in the network plot (Figure 4). Detailed information about network connections was presented in the supporting materials (Supplementary Tables S4–S7).

## Discussion

Our results reveal that the leaf and root microbiomes of spruce trees growing in a common garden show host genotype-specific structures. The presence of network modules consisting of phenotypic characters and taxonomically diverse plant-inhabiting microbes suggests that the status of plant-microbe interactions can be influenced by plant identity *per se*. Both the plant-inhabiting bacterial/archaeal and fungal community (phyllosphere and rhizosphere) were differentiated by the host species (Figures 3A,B). The plant effects were observed in two different aspects, involving phyllosphere versus soil and bacteria versus fungi.

The microbes inhabiting the phyllosphere and the rhizosphere were affected by plant species in different degrees. The clearest statistical evidence for this conclusion resulted from PCoA as shown in Figure 3, in which the samples of rhizosphere are grouped into clusters when considering both bacteria/archaea and fungi, whereas samples of phyllosphere had a relatively scattered distribution. It can be related to a habitat effect (plant compartment) as previously discussed (Bodenhausen et al., 2013; Coleman-Derr et al., 2016). The rhizosphere represents a highly more complex habitat than the phyllosphere. Since rhizosphere is surrounded by soil, into which plant roots continuously produce and secrete large and diverse amount of compounds (Knief et al., 2012; Zhalnina et al., 2018), a big proportion of root exudates will be accumulated in the soil, and, thus, they will have long-term effects on microbes by soil to rhizosphere. The plant-microbe interactions in the rhizosphere are affected through direct (root-microbe) and indirect (root-soil-microbe) pathways (Marschner et al., 2001). The rhizosphere community is influenced by plant genotype due to differences in the composition of root cell components and root exudates (Berg and Smalla, 2009; Aira et al., 2010). Root zone soils, on the other hand, having different physicochemical characteristics that result from a long-term accumulation of the effect from plant phenotypic traits, also exert strong effects on rhizosphere communities (Bartelt-Ryser et al., 2005; Ofek et al., 2014). The phyllosphere microbiota is only affected by the direct way of the plant. Moreover, the substances secreted by plants to leaf surface are limited and fluctuating, which cannot be accumulated, thereby presenting a short-term effect to epiphytes (Lindow and Brandl, 2003). In the common garden, plant effect is the root cause of these habitat differences by the very fact that they are faced with the consistent environmental stimulation.

The bacterial and fungal communities seem to differ in their dependence on the host plant with the fungal communities being subject to greater host specificity. As shown in Supplementary Figure S1, the effect of plants on fungi was up to 80%, whereas it was below 10% on bacteria. These different degrees of dependency associations are likely to be related to host evolutionary history, given the interaction between microbial community variance and host traits because more than 80% of living plant species are symbiotic with fungi, such as mycorrhizal fungi and fungal endophytes (Wang and Qiu, 2006). These symbionts help plants that are capable of conquering the land and thriving in natural ecosystems which are exposed to environmental stress throughout the evolutionary time (Field et al., 2015). The intimate partnerships have coevolved for almost 450 million years and have formed the basis for a high degree of host specificity in this long coevolutionary process (Raaijmakers et al., 2009). In addition, compared to bacteria/archaea, fungi are considered to utilize macromolecular polymers more directly and efficiently (Boer et al., 2005; Štursová et al., 2012), so fungi may be more easily influenced by biotic factors due to close association with plant species (Lange et al., 2014; Urbanová et al., 2015). To present the actual fact, host specificity is more common in fungi, such as arbuscular mycorrhizal fungi (AMF) (Sanders and Croll, 2010), in plant pathogens (van der Does and Rep, 2017), and endophytes (Saikkonen et al., 2004), than in bacteria. The majority of published studies are still based on the conventional wisdom that fungi are plant-defending mutualists. Yet, there were no direct quantitative comparisons in previous studies to indicate the high host specificity of fungi. The result found in this study is a solid evidence for this phenomenon.

There was a significant correspondence between tree phenotypic characters and microbial community phylogeny (Figures 2, 4a–d). The results imply the existence of specific relationships between the host tree and its microbiota. Such a viewpoint of correlated plant traits and phenotypic characters of taxonomically diverse microbes is expected to enhance our understanding of the “plant-microbe interaction” governed by the plant. Not all members of the plant microbiota show host genotype-specific identity in this study. This raises the possibility that at least some members of the microbial community adapt to plant genotypes. We think it is likely that the taxa connecting among phenotypic characters in the analyses have close affinities to species with diverse ecological functions. The traits related to plant resource uptake strategies, such as leaf nutrient concentrations (N and P), LMA, and photosynthetic capacity (Cond, photo, Ci and Trmmol), which are connected with bacterial and fungal taxa, are likely a result of the profound impact of leaf resource uptake strategies on leaf morphology and physiology (Wright et al., 2004; Osnas et al., 2013). A plant’s ability to garner resources is strongly influenced by its morphology. Plant physiology and morphology interact with each other to determine how growth depends on the availability of resources (Tilman, 1988). The association between the relative abundance of certain microbial taxa and the growth-related characters of host plant is likely to be related to the microbes directly influencing the growth of their hosts through resource uptake and exchange strategies. We can hypothesize that the traits of plant tissue morphology and physiology filter for a certain bacterial/fungal community composition by promoting or inhibiting the growth of different bacterial/fungal clades. Given the microbial succession on leaves, future studies will be required to fully track the large changes in the relative abundance of certain microbial taxa that are correlated to different leaf traits. Some bacteria connected with traits related to photosynthesis are pigmented, such as *Flavitalea*, in the phyllosphere. These microbes with pigments of chlorophylls, carotenoids, and bilins can store the energy of sunlight and deal with high solar radiation environments (Finkel et al., 2011; Atamna-Ismaeel et al., 2012a,b). If we assume a direct connection of leaf pigment contents and biochemistry processes within the leaves, our results could point toward a close interconnectivity of leaf-inhabiting bacteria with the tree host physiology. The possible causes of the correlation between leaf pigments and microbial members could be either a generally impaired or a stimulated plant defense under stressful conditions.

The interaction of various bacteria (and fungi) with plant leaves and roots suggest that there is an extensive bio-communication between them through the plant holobiont. For example, the genus *Methylotenera* is connected with the Cleaf and the MC, which are likely a result of the presence of one-carbon (C_1_) conversion processes in the phyllosphere and in the rhizosphere because the species of the genus *Methylotenera* are important functional types in environmental cycling of C_1_ compounds (Kalyuzhnaya et al., 2012). A prominent C_1_ source for phyllosphere microorganisms is methanol, which is a byproduct of pectin demethylation during plant cell-wall metabolism (Rastogi et al., 2013). It seems that the different plant compartments provide specific habitats that have very different characteristics (phyllosphere versus rhizosphere). Hence, the presence of the microorganisms may depend on the structure and characters of the habitat, for example, *Methylobacterium* contains most methylotrophic bacteria in the plant. The results show that in the rhizosphere, this genus also correlates to δ^13^C and Cleaf. *Methylobacterium* has been documented as a plant-selected member that can stimulate plant development and even seed germination (Venkatachalam et al., 2016). In this study, the genus *Paracoccus* is abundant in the phyllosphere, and most species of the genus are able to use a very wide range of simple and complex organic substrates, such as methylotrophic species on C_1_ compounds (Kelly et al., 2006). *Paracoccus* seems to be very suitable for survival on leaves in which nutrients are scarce. The C sources that have been identified on leaf surfaces include organic acids, carbohydrates, sugar alcohols, and amino acids (Vorholt, 2012). In previous studies, this genus has been documented as a key player in biogeochemical cycling (Dziewit et al., 2014). The genus *Rhizobium*, which seems to be another habitat-specific member, is abundantly present on plant leaf surfaces in this study. The *Rhizobium* species are N_2_-fixing bacteria that convert atmospheric N_2_ into ammonia for the plant (van Rhijn and Vanderleyden, 1995). The N_2_ fixation in the phyllosphere is the main mechanism for adding nitrogen (N) in terrestrial ecosystems and is a metabolically very expensive process (Abril et al., 2005; Reed et al., 2011). In this study, these high energy demands translate into a relatively high demand for adenosine triphosphate (ATP) and reduce power, such as the energy from sun and organic matter, which may account for a more strong correlation with leaf characteristics such as C, LL, Photo, Cleaf, and Cond, because the plants with higher values of C, LL, Photo, Cleaf, and Cond can usually provide more energy.

Fungal taxa are involved in saprotrophs, symbiotrophs, and plant and animal pathogens. For example, the genera of *Wilcoxina*, *Tuber*, *Suillus*, *Inocybe*, *Clavulina*, *Lyophyllum*, and *Sebacina* comprise a specific group of ascomyceteous fungi that form a distinct mycorrhizal association. The mycorrhizal hyphae network is an imperative gateway for photosynthetic C to belowground in the short term (Kaiser et al., 2015). Some species of these genera were previously known to be associated with promoters on plant growth (Prabhu et al., 1996; Tsavkelova et al., 2006). A portion of the fungal taxa is plant pathogens, such as *Ilyonectria*, *Protomyces*, *Ramularia*, and *Epicoccum*. They are mostly connected with water-related traits – SWC and Cleaf. There are also other microbes related to these traits. Water transport in leaf vasculature is a fundamental process affecting plant and microbe growth, ecological interactions, and ecosystem productivity (Gleason et al., 2018). The index, δ^13^C in leaf, is used to evaluate the water use efficiency of plants comprehensively. Further, saprotrophic, symbiotrophic, and pathogenic fungi can share niches and positively/negatively interact with different plant traits. For example, saprotroph (the genera *Rhizophlyctis* and *Ajellomyces*) and pathotroph (the genus *Chalara* and *Phacidium*) co-occurred with each other to compete for N elements. This finding suggests that positive co-occurrence networks included potential competitive interactions between plant-inhabiting fungi and plant traits. The co-occurrence analysis allows us to raise working hypotheses on correlations among different functional groups of bacteria/fungi with plant traits, and the observed patterns may be explained, at least partly, by suffering from the different host plant phenotypes.

All plant species in the common garden use the same major resources of light, water, CO_2_, and mineral nutrients. Phenotypic differences among the plant species ultimately arise from plant genotype. The leaves, stems, and roots vary between species in construction and in nutrient allocation. Plant as a holobiont governs its associated microbiota assembly both in aboveground and belowground (Mencuccini and Hölttä, 2010; Kaiser et al., 2015; Hacquard, 2016). As a result, by producing different phenotypes, plant genotypes eventually lead to the host specificity to its microbiota. However, different phenotypic characters have a selective effect on different functional groups of microorganisms. Different microorganisms also have different degrees of response to this selection of plants. In other words, phenotypic characteristics of the plant shape the composition of the microbes that it harbors. A similar understanding of the assembly of animal-associated microbiomes is known to be driven by ecologically important host attributes, such as diet (Muegge et al., 2011). These correlations between plant microbiota and host traits suggest that incorporating information on plant-microbe associations will improve our ability to understand plant functional biogeography and the drivers of variation in plant and ecosystem function. The stem-associated microbes are not measured by this study (unable to sample), which does not affect our conclusions for the strong associations presented here. This study provides a “snapshot” of plant-associated microbial communities in the current state. Long-term investigations of the effects of host traits on the structure and dynamics of its microbiota will be required to better understand the host specificity of the plant microbiota.

## Author Contributions

GZ, TC, WW, GL, and SL planned and designed the research. MW and CZ provided the help in sampling. XW, GZ, and HZ analyzed the data. YL and GZ conducted the experiments and wrote the manuscript. All authors were involved in revising the manuscript critically.

## Conflict of Interest Statement

The authors declare that the research was conducted in the absence of any commercial or financial relationships that could be construed as a potential conflict of interest.

## Footnotes

1 http://www.earthmicrobiome.org

2 http://www.qiime.org

3 http://www.arb-silva.de

4 http://unite.ut.ee/index.php
