# Supplementary material for: Plant Phenotypic Traits Eventually Shape Its Microbiota: A Common Garden Test
Source: Front Microbiol. 2018 Nov 6;9:2479. doi: 10.3389/fmicb.2018.02479 (PMC6232875; doi:10.3389/fmicb.2018.02479)

Supplementary Material

Article Title: Plant Phenotypic Traits Eventually Shape Its Microbiota：A Common Garden Test

Yunshi Li^1,2^, Xiukun Wu^1^, Tuo Chen^1,3^, Wanfu Wang^1,4^, Guangxiu Liu^1^, Wei Zhang^1^, Shiweng Li^5^, Minghao Wang^6^, Changming Zhao^6^, Huaizhe Zhou^7^, Gaosen Zhang^1*^

*** Correspondence:**

Gaosen Zhang

[gaosenzhang@hotmail.com](mailto:gaosenzhang@hotmail.com)

**Supplementary Figure S1** Individual bacterial/archaeal and fungal taxa at different taxonomic levels are sensitive to host attributes. The significant *p*<0.05 interactions with ANCOVA analysis were plotted. Line chart shows the total relative abundance (Mean±SE) of bacterial/archaeal (A,B) and fungal (C,D) taxa at multiple taxonomic levels with (B,D) and without (A,C) environmental factors. ‘LL’= leaf length; ‘LMA’ = leaf dry mass per area; ‘SWC’ = leaf water content; ‘Cleaf’ = leaf water storage capacity; ‘LTN’ = content of leaf total nitrogen; ‘P’ = phosphorous content in leaf; ‘K’ = potassium content in leaf; ‘δ^13^C’ = leaf δ^13^C values; ‘Cond’ = ‘stomatal conductance; ‘Photo’ = net photosynthetic rate; ‘Ci’ = intercellular carbon dioxide concentration; ‘Trmmol’ = transpiration rate.


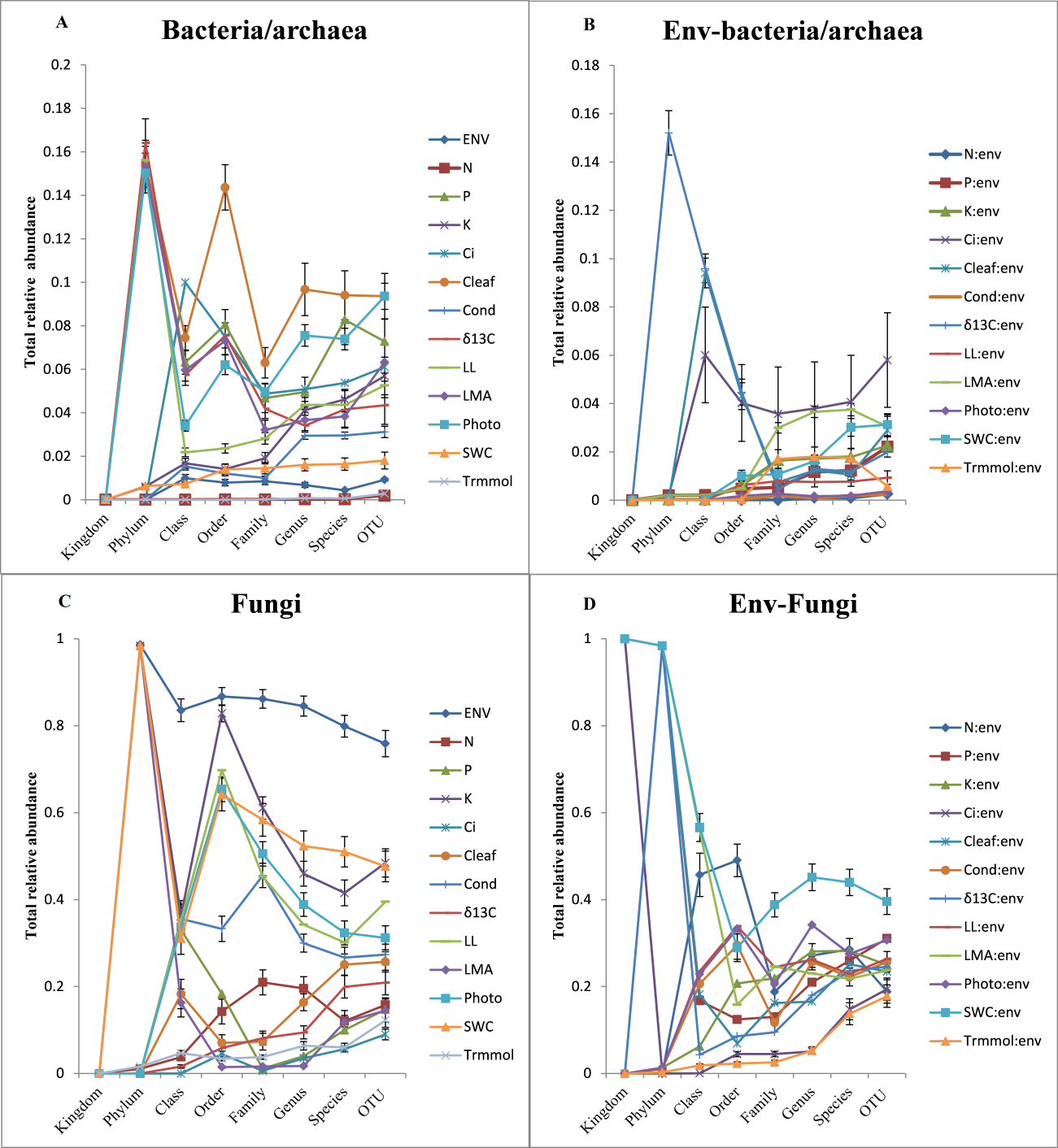


**Supplementary Figure S2** Heat map showing the correlations between bacterial/archaeal (A) and fungal (B) communities and host/soil attributes at genus-level taxonomy. The data were analyzed with ANCOVA analysis. In all, there are 479 bacterial/archaeal genera and 224 fungal genera, respectively, correlated with host/soil attributes. Only genera that represent ≥0.1% of the total population in at least one sample are included. ‘LL’= leaf length; ‘LMA’ = leaf dry mass per area; ‘SWC’ = leaf water content; ‘Cleaf’ = leaf water storage capacity; ‘LTN’ = content of leaf total nitrogen; ‘P’ = phosphorous content in leaf; ‘K’ = potassium content in leaf; ‘δ^13^C’ = leaf δ^13^C values; ‘Cond’ = ‘stomatal conductance; ‘Photo’ = net photosynthetic rate; ‘Ci’ = intercellular carbon dioxide concentration; ‘Trmmol’ = transpiration rate; ‘STN’ = content of soil total nitrogen; ‘TC’ = total carbon in soil; ‘OC’ = organic carbon in soil; ‘TDS’ = total dissolved solids in soil; ‘EC’ = soil electric conductivity; ‘Salinity’ = total soil salinity; ‘MC’ = soil moisture content.

**(A)**


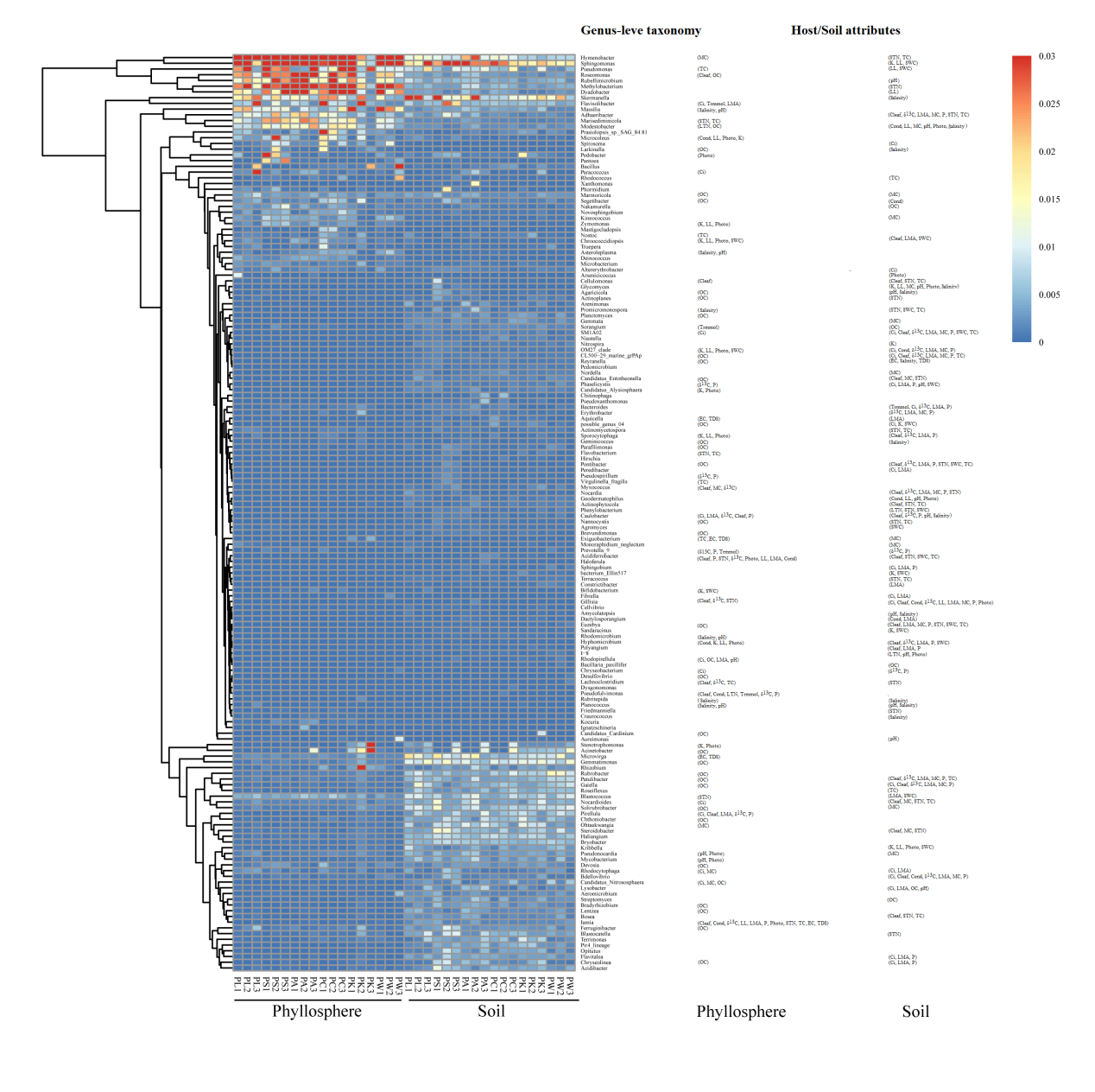


**(B)**


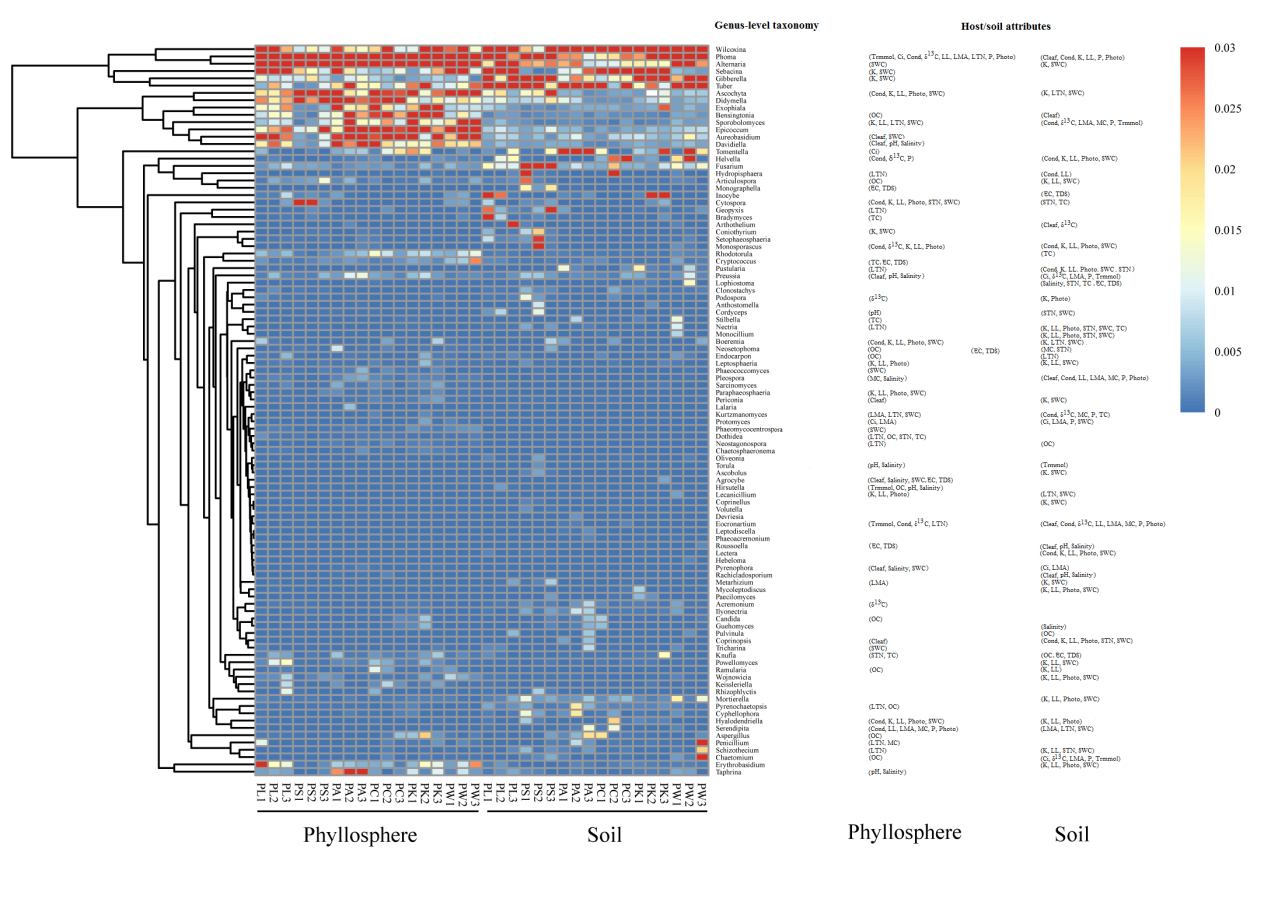

Supplement: Supplementary file 1 [file Table_1.DOCX]
